# Impact of Living Arrangements on Delirium in Older ED Patients

**DOI:** 10.3390/jcm14092948

**Published:** 2025-04-24

**Authors:** Andrea Russo, Sara Salini, Luigi Carbone, Andrea Piccioni, Francesco Pio Fontanella, Fiorella Ambrosio, Claudia Massaro, Davide Della Polla, Giuseppe De Matteis, Francesco Franceschi, Francesco Landi, Marcello Covino

**Affiliations:** 1 Geriatrics Department, Fondazione Policlinico Universitario A. Gemelli, IRCCS, 00168 Rome, Italy; andrea.russo1@policlinicogemelli.it (A.R.);; 2 Department of Emergency Medicine and Internal Medicine, Ospedale Fatebenefratelli Isola Tiberina, Gemelli-Isola, 00168 Rome, Italy; 3 Emergency Department, Fondazione Policlinico Universitario A. Gemelli, IRCCS, 00168 Rome, Italy; andrea.piccioni@policlinicogemelli.it (A.P.); marcello.covino@unicatt.it (M.C.); 4 Department of Internal Medicine, Fondazione Policlinico Universitario A. Gemelli, IRCCS, 00168 Rome, Italy; 5 Faculty of Medicine, Università Cattolica del Sacro Cuore, 00168 Rome, Italy

**Keywords:** frailty, comprehensive geriatric assessment, delirium, emergency department, living arrangement

## Abstract

**Background**: The purpose of this study is to assess how the socio–family demographic status of patients is related to the onset of delirium in a large cohort of older adults aged ≥65 years evaluated in the emergency department (ED) using a comprehensive geriatric assessment (CGA). **Methods**: This is a cross-sectional, observational, retrospective study conducted in the ED of a teaching hospital. We enrolled 2770 geriatric patients admitted to the ED from January 2019 to December 2023 and evaluated them using CGA. Clinical variables assessed in the ED were evaluated for associations with delirium onset and in-hospital mortality. **Results**: Delirium was statistically related to frailty statuses as measured via the Clinical Frailty Scale (CFS) (OR 1.47 [1.39–1.56]; *p* < 0.001). The occurrence of delirium was also associated with living arrangements: “living with other relatives” condition (OR 1.43 [1.12–1.83]; *p* = 0.004) and residence in a nursing home (OR 1.72 [1.30–2.31]; *p* < 0.001). In addition, compared to patients in emergency conditions (NEWS > 5), it emerges that patients with better clinical stability have a lower risk of developing delirium (NEWS 3–5 OR 0.604 [0.48–0.75]; *p* < 0.001—NEWS < 3 OR 0.42 [0.34–0.53]; *p* < 0.001). In-hospital mortality was associated with age, male sex, frailty status, clinical instability, and the onset of delirium in the ED. **Conclusions**: Delirium is a multifactorial and acute syndrome representing a negative prognostic factor of in-hospital mortality, especially in elderly patients. Independent of the clinical condition, the patient’s living arrangement could be of relevance to the onset of delirium in the ED. Early comprehensive geriatric assessments in the ED could allow the early detection of all predisposing risk factors, resulting in the timely implementation of supportive strategies to prevent the onset of delirium in EDs.

## 1. Introduction

Emergency departments (EDs) have faced a global challenge in recent years due to rapidly changing patient populations [1]. In particular, the increase in life expectancy has led to a worldwide increase in the number of elderly and comorbid patients accessing EDs [1,2,3].

This older population has undergone variations in socio–family variables over time; these patients often come from different and modified socio-demographic backgrounds: some live at home (with or without family and with or without assistance), and others in nursing homes [4,5].

Increased life expectancy rarely results in successful aging; more frequently, it results in cognitive and functional decline, often defined as “frailty”. Frailty leads to vulnerability and reduces resilience to stressors, resulting in an increased risk of adverse outcomes [6,7].

This older frail population is vulnerable to developing delirium, one of the most common complications for elderly persons with acute illnesses admitted to EDs [8,9].

Delirium is an acute neurological emergency syndrome defined by the transient and essentially reversible dysfunction of brain metabolism, and it has an acute or subacute onset and is clinically manifested by a wide range of neuropsychiatric anomalies [10]. It is a neurocognitive manifestation of an underlying medical abnormality (such as organ failure, infection, or drug effect).

Delirium is associated with adverse outcomes (increased length of stay, long-term functional and cognitive decline, higher healthcare costs, and increased mortality) [11,12].

Many risk factors have been identified, e.g., the use of new drugs, dementia, infection, dehydration, hypoxia, anemia, immobility, malnutrition, bladder catheter, hospitalization, being a nursing home resident, pain, sleep deprivation, visual and hearing impairment, and emotional stress [13,14]. Delirium is also known to be more frequent in patients living in frail conditions, with a correlation proportional to Clinical Frailty Scale scores [15,16].

A very significant role in the development of delirium is played by the socio–family environment. The presence of family and specific interventions involving the social network are crucial for the prevention of cognitive impairment and delirium [17,18,19].

However, delirium, in part due to the atypical symptoms of elderly patients, is often underdiagnosed, especially in EDs [20].

All these elements identify elderly patients with delirium as challenging to manage [21,22,23,24].

Given these assumptions, there is increasing scientific interest in the development of evaluating geriatric patients in a comprehensive way that is appropriate to their peculiar characteristics [25].

The Geriatric Emergency Department Guidelines recommend screening older adults during their ED visit for delirium, fall risk/safe mobility, frailty, and home safety needs [26,27].

A geriatric assessment could facilitate the implementation of personalized interventions that address the most appropriate care for individual patients [28]. Consequently, it is critical to understand risk factors and risk stratification approaches to streamline the identification of adverse outcomes, including delirium in elderly patients in EDs [29].

This study aims to assess, in a large cohort of adults aged ≥65 years who were evaluated in an ED via a comprehensive geriatric assessment (CGA), the relationship between the patient’s socio–family demographic status and the occurrence of delirium and the all-cause in-hospital mortality.

## 2. Materials and Methods

### 2.1. Study Design

This is a cross-sectional observational retrospective study conducted in the ED of a teaching hospital with an annual attendance of about 75,000 patients, with 87% being adults. We analyzed 2770 geriatric patients admitted to the ED from January 2019 to December 2023 and evaluated with CGA.

The inclusion criteria are as follows:

- Patients with age ≥ 65 years;
- Presence of comprehensive geriatric assessment;
- Hospitalization following the ED visit.

The exclusion criteria are as follows:

- Age < 65 years (*n* = 428);
- Death in the ED (*n* = 6);
- Discharge (*n* = 1345);
- Transfer to other hospitals (*n* = 3262).

### 2.2. Study Variables

For all patients included in the study cohort, demographic characteristics (age and sex) and vital parameters were reviewed. In the case of several measurements, the first values were considered. Each patient had a National Early Warning Score (NEWS) [30] assessment upon ED admission. This score identifies three levels related to the clinical severity of the patient: low (code green, score 1 to 4), medium (code yellow, score 5 to 6), and high (code red, score ≥ 6).

A geriatric multidimensional assessment was performed for all patients. During the CGA, the Clinical Frailty Scale score [16,31] was calculated. Based on the CFS value, patients were divided into fit (CFS ≤ 3), mildly frail (CFS > 3 or ≤ 6), and frail (CFS > 6).

The delirium condition was investigated using the Brief Confusion Assessment Method (positive—presence of delirium; negative—absence of delirium) [32].

Vigilance status was calculated using the Richmond Agitation-Sedation Scale (hyperactive type 1–4; hypoactive type −1/−5) [33].

The dementia condition was estimated using the Clinical Dementia Rating, a numeric scale used to quantify the severity of symptoms of dementia [34].

Comorbidities were recorded, including hypertension, ischemic heart disease, heart failure, chronic respiratory obstructive disease (COPD), peripheral vascular disease, dementia, diabetes, chronic kidney disease, malignancy, and leukemia/lymphoma. Overall, the comorbidity status was assessed via the Charlson Comorbidity Index [35].

All patients had a blood sampling for routine laboratory testing.

Socio-demographic data were investigated at triage and reported to the informatic system.

The living arrangements of the patients were categorized into four groups: patients living alone (with limited or no support); patients living with their partner (including patients with continuous support from the partner); patients living with their relatives (including patients with full continuous support from relatives different from a partner); patients living in a residential institution (nursing home, senior living facilities, and senior foster home).

### 2.3. Outcome Measures

The primary endpoint of the study was the association between the examined risk factors and the prevalence of delirium in the ED. The secondary outcome was the association between examined risk factors and all-cause hospital mortality.

### 2.4. Statistical Analysis

Categorical variables are presented as absolute numbers and percentages; continuous variables are presented as a median (interquartile range). Categorical variables were statistically compared using the chi-square test or Fisher’s exact test as appropriate. Continuous variables were compared using the Mann–Whitney U test or, in the case of three or more groups, the Wilcoxon ANOVA median test. Post hoc analysis was carried out using the Bonferroni test for multiple comparisons.

Significant factors at univariate analysis were entered into a univariate and subsequently multivariate logistic Cox model to identify independent risk factors for the defined endpoints. The variable of interest for the study (living arrangement) was forced in all multivariate models.

In the case of combined variables, such as single comorbidities and the Charlson Index, the composing factors were excluded from the analysis to avoid parameter overestimation and instability. Similarly, the factors already considered in the analysis endpoint (such as RASS and delirium) were excluded from the multivariate model. Finally, we excluded variables with fewer than 10 expected events from the model to avoid model instability.

According to these premises, a multivariate Cox regression model was used to determine the adjusted risk of delirium occurrence in the ED. Similarly, a multivariate Cox regression model was used to determine the adjusted risk of in-hospital death for patients (including in the analysis the delirium occurrence as a categorical variable). Survival curves were calculated according to the Kaplan–Meier method. Cox regression analysis results were reported as a Hazard Ratio (HR) [95% confidence interval].

All *p* values were 2-sided, with a significance threshold set at 0.05 and corrected in the case of multiple group comparisons. The study’s analysis was conducted using SPSS version 25 (IBM, Armonk, NY, USA).

## 3. Results

### 3.1. Study Population

In the study period, 2770 older adults were screened for the Multidimensional Geriatric Assessment. The median age of the sample was 84 years [77–89], and 1188 (42.9%) patients were male.

Table 1 summarizes the characteristics of patients divided according to socio-demographic patterns. The living alone group consisted of 553 (19.96%) patients; the living with partner group had 692 (24.98%) patients; the living with relatives group had 1014 (36.60%) patients; the nursing home group had 511 (18.44%) patients.

According to frailty as determined by CFS, the fit group consisted of 227 (8.2%) patients; the mildly frail group had 1103 (39.8%) patients, and the frail group had 1440 (52.0%) patients.

The most frequent presenting symptoms recorded in all groups were fever (31%), dyspnea (22.8%), trauma (16.7%), and disorientation (13.3%). In a considerable percentage (44.8%), the symptoms were present for more than 24 h before ED access.

Regarding clinical severity upon ED presentation, 437 (15.8) patients had a NEWS > 5, 1041 (37.6) a NEWS 3–5, and 1292 (46.6) a NEWS < 3.

The most frequent diagnoses at discharge from the hospital were infectious diseases (30.9%), followed by cardiovascular diseases (23.2%) and metabolic disorders (15.8%).

Overall, 1045 (37.7) of the patients in the study cohort developed delirium during the ED stay. Delirium incidence was different in patients according to their living arrangements. The most affected patients were those in the group living with relatives (38.1%) compared to those living in nursing homes (24.3%), those living with a partner (20.0%), and those living alone (17.6%) (*p* < 0.001).

Regarding the in-hospital mortality, 147 (28.8%) nursing home patients died during hospitalization, compared to 224 (22.1%) patients living with relatives, 105 (15.2%) patients living with a partner, and 68 (12.3%) patients living alone (*p* < 0.001). The overall length of stay was slightly higher among the groups, with the longest LOS observed in patients living in nursing homes and the shortest LOS observed in patients living with their partner (*p* = 0.02) (Table 1).

The post hoc analysis revealed that the patients living alone had a similar age compared to patients living with their relatives and were significantly older than the patients living with their partner or those living in a nursing home. Similarly, the analysis evidenced that the patients living in a nursing home were significantly frailer than the other three groups, according to CFS.

### 3.2. Delirium Occurrence in the ED

In the study cohort, 1045 (37.7) patients experienced delirium during the ED stay. Table 2 summarizes the clinical characteristics of enrolled patients according to delirium conditions.

The occurrence of delirium is more prevalent in patients residing in nursing homes (49.7) and patients living with relatives (39.3). Delirium is less frequent in patients living alone (33.3) or those living with a partner (30.2) (*p* < 0.001).

The condition of frailty was found to be correlated with the occurrence of delirium (*p* < 0.001). Delirium was also related to age (*p* < 0.001), male sex (*p* < 0.001), pre-existing dementia (*p* < 0.001), and NEWS (*p* < 0.001).

The occurrence of delirium appeared to be associated with all-cause mortality (*p* < 0.001).

Table 3 summarizes the odds ratios for delirium concerning the variables of interest.

Delirium was strongly related to the frailty condition (OR 1.47 [1.39–1.56]; *p* < 0.001).

The occurrence of delirium was also associated with living arrangements. Indeed, it was associated with the “living with other relatives” condition (OR 1.43 [1.12–1.83]; *p* = 0.004) and residence in a nursing home (OR 1.72 [1.30–2.31]; *p* < 0.001).

In addition, compared with patients in emergency conditions (NEWS > 5), it emerges that patients with better clinical stability have a lower risk of developing delirium (NEWS 3–5 OR 0.604 [0.48–0.75]; *p* < 0.001—NEWS < 3 OR 0.42 [0.34–0.53]; *p* < 0.001).

Regarding discharge diagnosis, only the worsening of cognitive impairment was associated with the development of delirium (OR 2.31 [1.74–3.06]; *p* < 0.001).

There were no correlations with sex (OR 0.99 [0.83–1.19]; *p* = 0.946) and comorbidities (OR 1.001 [0.97–1.04]; *p* = 0.948). No correlation was equally found with the total ED time (*p* = 0.22).

In the study sample, 544 (19.6) patients died during hospitalization; Table 4 summarizes the comparison of surviving vs. deceased patients during the hospitalization (all-cause death).

As expected, the degree of frailty was found to be correlated with mortality: Among the deceased patients, 14 (2.6) were fit, 122 (22.4) had moderate frailty, and 408 (75) were frail (*p* < 0.001). The occurrence of delirium appeared to be associated with all-cause mortality: 308 (56.6) of the deceased patients experienced delirium occurrence during the ED stay (*p* < 0.001). All-cause mortality was found to be correlated with patients living in a nursing home (28.8) and those living with family relatives (22.1) (*p* < 0.001).

Table 5 summarizes the multivariate Cox regression model for the variables associated with in-hospital death in the study cohort.

**Figure 1:**
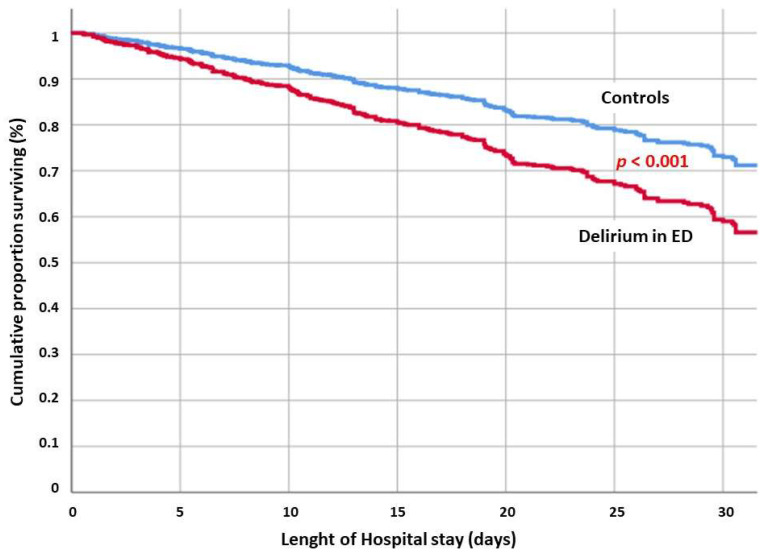
Adjusted cumulative survival for patients with delirium occurrence in the ED compared to controls.

Once adjusted for significant confounders, the in-hospital mortality was found to be correlated with age (HR 1.03 [1.02–1.04]; *p* < 0.001), male sex (HR 1.51 [1.27–1.81]; *p* < 0.001), frailty as expressed by CFS (HR 1.44 [1.34–1.55]; *p* < 0.001), and delirium occurrence in the emergency department (HR 1.57 [1.30–1.88]; *p* < 0.001) (Figure 1). As expected, compared with critical patients (NEWS > 5), patients with better clinical stability have a lower risk of death (NEWS < 3 HR 0.59 [0.47–0.76]; *p* < 0.001).

In-hospital mortality was also correlated with total ED time (HR 1.01 [0.99–1.01]; *p* < 0.001).

Interestingly, there was no association between in-hospital death and comorbidities (HR 1.02 [0.97–1.06]; *p* = 0.355).

No correlation was found between in-hospital mortality and living arrangements.

To stratify the interactions between delirium, frailty, and comorbidities, a multivariate Cox regression model was obtained, including delirium, severity at hospital admission (based on the NEWS), Charlson Comorbidity Index, CFS, and age. The analysis revealed that, once adjusted for these covariates, the occurrence of delirium in the ED increased the risk of in-hospital mortality (HR 1.81 [1.53–2.15], *p* < 0.001) (Figure 1).

## 4. Discussion

The first finding of this study is the high prevalence of delirium in emergency departments (n = 983, 35.5%), which is higher than the upper limit reported in the literature. The evidence reports that delirium occurs in 6% to 36% of elderly patients in the emergency department (ED) [36]. Epidemiologically, age is a variable associated with delirium. Several studies show that delirium is age-dependent, a finding confirmed by our study. A recent systematic review study showed advanced age as a risk factor for delirium [13].

In our population, frail patients had a significantly higher probability of developing delirium during their ED stay than less frail or non-frail patients after adjusting for confounders [37]. Moreover, frailty appears to be a better predictor of ED-induced delirium than comorbidity. This is because frail elderly people are less resilient in the face of stressors such as the ER environment or acute clinical distress.

The observation that frailty is associated with delirium deserves comment. A recent single-center study explored the relationship between frailty and delirium in patients admitted to an acute geriatric unit [38,39]. The reason behind this correlation may lie not only in reduced resilience but also in the frail patient’s objective difficulty in answering the questions asked because of a previous objective [40].

By consequence, in elderly patients with frailty, different and more specific multidimensional and holistic approaches to delirium screening may be needed [41].

One expected finding is clinical instability at the time of arrival in the emergency department. Our data show that the risk of developing delirium is higher in patients who arrive in the ED with more critical conditions (NEWS > 5) [13,42,43].

An interesting finding, on the other hand, concerns the onset of delirium and the time spent in the emergency room. Compared to data in the literature, our work shows that the onset of delirium is independent of the time spent in the ED [44].

The most important and unexpected finding from our analysis concerns socio–family demographic background. Our work shows that patients from nursing homes are at higher risk of developing delirium, followed by those with a well-represented family background. In contrast, patients living alone or with one person seem to have a lower risk.

Some data on nursing home residents confirm our results; other data do not confirm this as a risk factor for new-onset delirium [45]. Such differences in results could be explained by regional differences or differences in methodology.

Socio–family variables involving living arrangements can be explained by the fact that patients living alone or with a partner are more self-sufficient and therefore less frail. In contrast, patients who are residents in nursing homes are generally patients with a high degree of frailty and comorbidities, often suffering from dementia or hypomobility syndrome.

Another interesting finding is about patients with a good family background who, from our analysis, are found to be most at risk. What we can call “affective sensory deprivation” probably occurs in these patients. They are used to being surrounded by two or more family members and living in more social settings. At admission to the emergency room, the deprivation of this stimulus could be a determinant in the onset of delirium.

Sensory deprivation occurs when an individual receives a stimulus that is reduced or below the threshold of normal; this typically occurs when the patient is placed in isolation, as in the case of changes in the environment due to admission to the emergency room. While in isolation, there can be a reduction in the quantity and quality of stimuli and restrictions on social interactions [46].

The association between the onset of delirium and “other sensory deprivation” was codified better, specifically hearing and visual sensory deprivation [47].

Our study draws attention to justifying the results obtained from another sensory system, interoception, which allows us to understand and feel what is happening inside our bodies.

Interoceptive dysfunction is now identified as an important part of several mental health conditions, including anxiety disorders, mood disorders, eating disorders, addiction disorders, and somatic symptom disorders, and it could be a possible substrate of the onset of delirium [48,49].

High levels of interoceptive awareness are linked to the development of many important skills, including self-awareness, self-regulation, problem-solving, decision-making, flexible thinking, social awareness, empathy, and perspective-taking [50]; in contrast, individuals with reduced interoceptive awareness have difficulty developing these skills, with significant consequences for health, well-being, and social interactions [50].

Indeed, some studies suggest that cognitive and physical changes occur in adulthood (over 65), which may be supported by changes in interoception. For example, increased risk of dehydration; social and emotional difficulties, including poor emotion recognition; and risky decision-making have been linked to or supported by interoception [51].

Regarding the in-hospital mortality outcome, our data suggest that delirium and age, frailty, the male gender, and NEWS in the emergency department are negative prognostic factors [52,53].

A new model of comprehensive geriatric assessment is critical for the identification and stratification of frailty [54] and the early identification of delirium in the emergency department [37], both of which are widely used in prognostic assessments in multiple acute-phase conditions [55,56,57].

Given these premises, a multidimensional and holistic approach is fundamental to the management of reversible risk factors of the onset of delirium in EDs [14,29].

Among the intervention strategies, those that act on certain risk factors are particularly effective, specifically cognitive impairment, sleep deprivation, immobility, visual impairment, hearing impairment, and dehydration. Non-pharmacological interventions for these factors are particularly effective [58]. Other studies have focused on strategies that can be implemented within the emergency department. In this context, the optimization of hemodynamics and oxygenation, pain management, hydration, and nutritional support is key.

Also, in the emergency room, it would be desirable to educate healthcare providers on early detection and non-pharmacological treatment [59].

Tailored interventions should include early mobilization, physical therapy, reorientation, cognitive stimulation, drug reviews, environmental stimulation, the avoidance of sensory deprivation, pain control, restraint use avoidance, and family participation [60].

Regarding our data, having considered that patients who are deprived of the closeness of family members are at greater risk of developing delirium, it would be appropriate to implement non-pharmacological intervention strategies involving family members, especially when the patient comes from a multi-member household [47].

In this regard, a reduction in the incidence of delirium in the emergency department is reported in the literature when intervention strategies involving the family are implemented. Family members provided information on orientation–memory clues, including the use of a family member’s voice, increased family visits or increased flexibility, or the use of virtual communication [61,62].

### Study Limitations and Strengths

This study has some limitations. As this is a retrospective, observational, case–control study, potential confounders may not have been addressed. Prospective longitudinal studies and possibly randomized controlled clinical trials are needed to confirm our findings. Additionally, this is a single-center observational study, so the results are not generalizable to all emergency departments. A selection bias may have occurred due to the exclusion of patients who were hospitalized in another hospital from the analysis.

This study only includes patients who received a CGA. The exclusion of patients without CGA may have potentially resulted in the misestimation of the prevalence of delirium.

Finally, since only the patients who received a full geriatric assessment were included, the study cohort may not be representative of the entire population of elderly people coming to the ED.

The prevalence of delirium and other geriatric syndromes was assessed only during the emergency room stay. There are no data collected on the prevalence of delirium during hospital stay.

However, although we tried to adjust our analysis for all potential confounders, we cannot exclude unmeasured, residual, and time-varying confounding factors that could affect the results.

The study’s strengths include the large sample size and the adjustment of the analysis for several clinical confounders.

## 5. Conclusions

Delirium is a multifactorial and acute syndrome that represents a prognostic factor for in-hospital mortality, particularly in older patients. Consequently, it is mandatory to implement all possible diagnostic and therapeutic strategies, as well as adjust modifiable risk factors, upon ED admission. Independent of the clinical conditions, the residential arrangement of the patient could be relevant for delirium occurrence in the ED.

Early comprehensive geriatric assessment in the ED could allow the early investigation and diagnosis of predisposing risk factors, resulting in the timely implementation of strategies to prevent the onset of delirium in the ED. Accordingly, the first step in improving the prognosis of frail elderly patients could be to encourage cooperation between geriatricians and emergency medicine physicians. Such cooperation would involve both sharing clinical assessments and therapy planning and improving the emergency physician’s training on the importance of geriatric syndromes (e.g., with webinars, surveys, etc.).

In the management of frail patients in EDs, moreover, a multidisciplinary team could be essential, encouraging the introduction of dedicated figures (physiotherapist, occupational therapist, case manager, etc.) in order to prevent the onset of complications (delirium, falls, infections, bedsores, etc.).

Other strategies could include involving family members in patient care and, where this is not possible, implementing alternative strategies (e.g., including professional figures such as a psychologist). Encouraging the presence of volunteers or caregivers could improve interventions on risk factors and the use of non-pharmacological therapies.

Further studies are needed to evaluate if these strategies could be successful in preventing delirium in the ED and ultimately reduce in-hospital mortality.

## Figures and Tables

**Table 1:** Clinical characteristics of enrolled patients according to their living arrangements.

Variable	All n = 2770	Living Alone n = 553	Living with Partner n = 692	Living with Other Relatives n = 1014	RSA n = 511	*p*
Age	84 [77–89]	85 [80–90]	80 [73–85]	86 [80–91]	84 [77–90]	<0.001
Sex (male)	118 (42.9)	213 (38.5)	473 (68.4)	332 (32.7)	170 (33.3)	<0.001
Clinical scales						
Clinical Frailty Scale	7 [5–8]	6 [4–7]	6 [5–7]	7 [5–8]	7 [7–8]	<0.001
Fit (CFS 1–3)	227 (8.2)	92 (16.6)	57 (8.2)	73 (7.2)	5 (1)	
Mildly frail (CFS 4–6)	1103 (39.8)	277 (50.1)	303 (43.8)	408 (40.2)	115 (22.5)	<0.001
Frail (CFS 7–9)	1440 (52)	184 (33.3)	332 (48)	533 (52.6)	391 (76.5)	
Clinical Dementia Rating	1 [0–2]	0 [0–2]	0 [0–2]	1 [0–1]	2 [1–3]	<0.001
RASS Value	0 [−1–0]	0 [0–0]	0 [−1–0]	0 [−1–0]	0 [−2–0]	<0.001
RASS-ABS	0 [0–1]	0 [0–0]	0 [0–1]	0 [0–1]	1 [0–2]	<0.001
Charlson Comorbidity Index	5 [4–7]	5 [4–7]	5 [4–7]	5 [4–7]	5 [4–7]	0.06
Presentation symptoms						
Trauma	463 (16.7)	139 (25.1)	104 (15)	168 (16.6)	52 (10.2)	<0.001
Dyspnea	631 (22.8)	105 (19)	137 (19.8)	215 (21.2)	174 (34.1)	<0.001
Fever	858 (31)	139 (25.1)	225 (32.5)	165 (26.1)	229 (44.8)	<0.001
Cough	103 (3.7)	19 (3.4)	38 (5.5)	34 (3.4)	12 (2.3)	0.03
Chest pain	92 (3.3)	28 (5.1)	26 (3.8)	37 (3.6)	1 (0.2)	<0.001
Syncope	259 (9.4)	77 (13.9)	69 (10)	102 (10.1)	11 (2.2)	<0.001
Abdominal pain	50 (9)	77 (11.1)	110 (10.8)	31 (6.1)	268 (9.7)	0.01
Vomit	227 (8.2)	53 (9.6)	62 (9)	79 (7.8)	33 (6.5)	0.24
Pain (any)	154 (5.6)	23 (4.2)	33 (4.8)	76 (7.5)	22 (4.3)	0.01
Disorientation	369 (13.3)	96 (17.4)	97 (14)	142 (14)	34 (6.7)	<0.001
Decreased consciousness	278 (10)	36 (6.5)	57 (8.2)	99 (9.8)	86 (16.8)	<0.001
Focal neurologic deficits	322 (11.6)	64 (11.6)	83 (12)	121 (11.9)	54 (10.6)	0.8
Malaise/fatigue	349 (12.6)	82 (14.8)	102 (14.7)	142 (14)	23 (4.5)	<0.001
Symptom onset (>24 h)	1242 (44.8)	226 (40.9)	360 (52)	444 (43.8)	212 (41.5)	<0.001
NEWS upon ED Admission						
NEWS > 5	437 (15.8)	77 (13.9)	74 (10.7)	182 (17.9)	104 (20.4)	
NEWS 3–5	1041 (37.6)	201 (36.6)	250 (36.1)	410 (40.4)	180 (35.2)	<0.001
NEWS < 3	1292 (46.6)	275 (49.7)	368 (53.2)	422 (41.6)	227 (44.4)	
Vitals						
Heart rate	85 [75–98]	85 [75–100]	85 [75–100]	84 [74–95]	86 [75–102]	<0.001
Systolic blood pressure	130 [110–150]	138 [118–155]	130 [110–149]	130 [110–150]	120 [100–139]	<0.001
Diastolic blood pressure	74 [64–85]	76 [65–87]	76 [67–85]	75 [64–86]	70 [60–80]	<0.001
SpO2	96 [94–98]	96 [95–98]	96 [94–98]	96 [94–98]	94 [91–97]	<0.001
Body temperature	36.2 [36–36.8]	36.2 [36–36.7]	36.1 [36–36.7]	36 [36–36.7]	36.5 [36–37.1]	<0.001
Laboratory values						
Serum creatinine	0.96 [0.73–1.47]	0.96 [0.74–1.41]	0.94 [0.74–1.5]	0.97 [0.74–1.48]	0.89 [0.66–1.53]	0.21
Glucose	121 [102–152]	117 [99–141]	121 [102–150]	124 [105–162]	119 [99–153]	0.001
NTproBNP	2550 [995–8059]	1692 [864–8038]	2131 [504–6180]	2668 [1270–8059]	3469 [1240–11,097]	0.004
Hb	12 [10.5–13.4]	12.3 [10.7–13.7]	12.3 [10.6–13.7]	12 [10–13.6]	11.7 [10–12.7]	<0.001
WBC	9.9 [7.3–13.4]	9 [6.6–11.9]	9.5 [7–13.4]	10.1 [7.6–13.8]	10.9 [8.1–15.6]	<0.001
PLT	237 [172–317]	238 [172–318]	227 [169–290]	231 [171–307]	264 [194–363]	<0.001
C-reactive protein	35 [9–111]	16 [2–78]	29 [7–110]	28 [9–81]	78 [31–150]	<0.001
Procalcitonin	0.14 [0.05–0.58]	0.11 [0.05–0.29]	0.14 [0.05–0.61]	0.13 [0.05–0.43]	0.22 [0.07–2.37]	<0.001
Clinical history						
Ischemic heart disease	247 (8.9)	34 (6.1)	79 (11.4)	89 (8.8)	45 (8.8)	0.01
Congestive heart failure	801 (28.9)	145 (26.2)	148 (21.4)	311 (30.7)	197 (38.6)	<0.001
Atrial fibrillation	519 (18.7)	115 (20.8)	109 (15.8)	201 (19.8)	94 (18.4)	0.09
Peripheral vascular disease	95 (3.4)	17 (3.1)	29 (4.2)	35 (3.5)	14 (2.7)	0.5
Previous stroke	308 (11.1)	41 (7.4)	70 (10.1)	125 (12.3)	72 (14.1)	0.002
Dementia	981 (35.4)	182 (32.9)	206 (29.8)	340 (33.5)	253 (49.5)	<0.001
COPD ^#^	348 (12.6)	63 (11.4)	68 (9.8)	133 (13.1)	84 (16.4)	0.005
Diabetes	380 (13.7)	68 (12.3)	102 (14.7)	146 (14.4)	64 (12.5)	0.46
Liver chronic disease	143 (5.2)	24 (4.3)	42 (6.1)	48 (4.7)	29 (5.7)	0.46
Chronic kidney disease	451 (16.3)	95 (17.2)	94 (13.6)	168 (16.6)	94 (18.4)	0.12
Malignancy	414 (14.9)	70 (12.7)	162 (23.4)	152 (15)	30 (5.9)	<0.001
Palliative care	40 (1.4)	0 (0)	12 (1.7)	22 (2.2)	6 (1.2)	0.006
Discharge diagnosis						
Cardiovascular disease	643 (23.2)	152 (27.5)	148 (21.4)	276 (27.2)	67 (13.1)	
Fall/trauma	166 (6)	73 (13.2)	40 (5.8)	40 (3.9)	13 (2.5)	
Infectious disease	857 (30.9)	117 (21.2)	187 (27)	265 (26.1)	288 (56.4)	<0.001
Cognitive impairment	360 (13)	79 (14.3)	101 (14.6)	143 (14.1)	37 (7.2)	
Malignancy	305 (11)	41 (7.4)	132 (19.1)	116 (11.4)	16 (3.1)	
Metabolic disorders	439 (15.8)	91 (16.5)	84 (12.1)	174 (17.2)	90 (17.6)	
Outcomes						
Death	544 (19.6)	68 (12.3)	105 (15.2)	224 (22.1)	147 (28.8)	<0.001
Delirium	1045 (37.7)	184 (17.6)	209 (20.0)	398 (38.1)	254 (24.3)	<0.001
Total ED time (hours)	48 [27–74]	50 [27–77]	44 [26–72]	51 [29–77]	46 [27–71]	<0.001
LOS ^£^ (days)	10 [6–16]	10 [6–17]	9 [6–15]	10 [6–16]	11 [7–19]	0.02

^#^ Chronic obstructive pulmonary disease; ^£^ length of hospital stay.

**Table 2:** Clinical characteristics of enrolled patients according to the delirium condition.

Variable	Delirium n = 1045	No Delirium n = 1725	*p*
Age	85 [78–90]	84 [77–89]	<0.001
Sex (male)	410 (39.2)	778 (45.1)	<0.001
Living Arrangements (%in rows)			
Living alone	184 (33.3)	369 (66.7)	
Living with a partner	209 (30.2)	483 (69.8)	<0.001
Living with relatives	398 (39.3)	616 (60.7)	
Nursing home resident	254 (49.7)	257 (50.3)	
Clinical scales			
Clinical Frailty Scale	7 [6–8]	6 [5–7]	<0.001
Fit (CFS 1–3)	38 (3.6)	189 (11)	
Mildly frail (CFS 4–6)	316 (30.2)	787 (45.6)	<0.001
Frail (CFS 7–9)	691 (66.1)	749 (43.4)	
Clinical Dementia Rating	2 [1–3]	0 [0–1]	<0.001
Charlson Comorbidity Index	5 [4–7]	5 [4–7]	0.78
NEWS > 5	225 (21.5)	212 (12.3)	
NEWS 3–5	412 (39.4)	629 (36.5)	<0.001
NEWS < 3	408 (39.0)	884 (51.2)	
Presentation symptoms			
Dyspnea	226 (21.6)	405 (23.5)	0.140
Fever	326 (31.2)	532 (30.8)	0.438
Cough	27 (2.6)	76 (4.4)	0.08
Chest pain	15 (1.4)	77 (4.5)	<0.001
Syncope	76 (7.3)	183 (10.6)	0.002
Abdominal pain	59 (5.6)	209 (12.1)	<0.001
Vomit	70 (6.7)	157 (9.1)	0.014
Pain (any)	69 (6.6)	85 (4.9)	0.039
Disorientation	191 (18.3)	178 (10.3)	<0.001
Decreased consciousness	179 (17.1)	99 (5.7)	<0.001
Focal neurologic deficits	148 (14.2)	174 (10.1)	0.001
Malaise/fatigue	104 (10.0)	245 (14.2)	0.001
Symptom onset (>24 h)	417 (39.9)	825 (47.8)	<0.001
Vitals			
Heart rate	85 [74–100]	85 [75–97]	0.43
Systolic blood pressure	122 [106–145]	130 [110–150]	<0.001
Diastolic blood pressure	73 [60–85]	75 [65–86]	0.016
SpO_2_	95 [92–97]	96 [94–98]	<0.001
Body temperature	36.3 [36–36.9]	36.1 [36–36.7]	<0.001
Laboratory values			
Serum creatinine	0.95 [0.70–1.5]	0.96 [0.73–1.45]	0.849
Glucose	121 [102–153]	120 [102–151]	0.836
NTproBNP	2504 [931–8123]	2668 [1049–7771]	0.979
Hb	12 [10.7–13.7]	12 [10.4–13.2]	0.023
WBC	10.6 [7.8–14.7]	9.5 [7.2–12.6]	<0.001
PLT	236 [169–314]	237 [178–318]	0.365
C-reactive protein	47 [12.8–135.9]	28.7 [7.6–93.4]	<0.001
Procalcitonin	0.19 [0.05–0.69]	0.12 [0.05–0.50]	0.002
Clinical History			
Ischemic heart disease	68 (6.5)	179 (10.4)	<0.001
Congestive heart failure	314 (30.0)	487 (28.2)	0.164
Atrial fibrillation	192 (18.4)	327 (19.0)	0.371
Peripheral vascular disease	39 (3.7)	56 (3.2)	0.282
Previous stroke	155 (14.8)	153 (8.9)	<0.001
Dementia	517 (49.5)	464 (26.9)	<0.001
COPD ^#^	114 (10.9)	234 (13.6)	0.023
Diabetes	165 (15.8)	215 (12.5)	0.008
Liver chronic disease	13 (1.2)	43 (2.5)	0.015
Chronic kidney disease	182 (17.4)	269 (15.6)	0.114
Malignancy	112 (10.7)	302 (17.5)	<0.001
Palliative care	11 (1.1)	29 (1.7)	0.118
Cardiovascular disease	210 (20.1)	433 (25.1)	
Fall/trauma	50 (4.8)	116 (6.7)	
Infectious disease	358 (34.3)	499 (28.9)	<0.001
Cognitive impairment	194 (18.6)	166 (9.6)	
Malignancy	74 (7.1)	231 (13.4)	
Metabolic disorders	159 (15.2)	280 (16.2)	
Death	308 (29.5)	236 (13.7)	<0.001
Total ED time (hours)	48 [28–73]	48 [27–75]	0.959
LOS ^£^ (days)	11 [6–17]	10 [6–16]	0.267

^#^ Chronic obstructive pulmonary disease; ^£^ length of hospital stay.

**Table 3:** Multivariate logistic regression model for the occurrence of delirium in the ED. The overall model’s chi-square was 316.75; −2Log-likelihood was 3354.65.

Variable	Odds Ratio	Wald	*p*-Value
Age	1.01 [1.01–1.02]	9.33	0.002
Sex (male)	0.99 [0.83–1.19]	0.005	0.946
Clinical scales			
CFS	1.47 [1.39–1.56]	171.54	<0.001
CCI	1.001 [0.97–1.04]	1.75	0.948
NEWS > 5	Reference category	56.24	
NEWS 3–5	0.604 [0.48–0.75]	17.67	<0.001
NEWS < 3	0.42 [0.34–0.53]	54.41	<0.001
Living Arrangements			
Living alone	Reference Category	8.94	
Living with partner	0.94 [0.71–1.24]	0.18	0.670
Living with relatives	1.43 [1.12–1.83]	8.37	0.004
Nursing home resident	1.72 [1.30–2.31]	13.9	<0.001
Discharge diagnosis			
Cardiovascular disease	Reference Category	78.11	
Fall/trauma	0.95 [0.63–1.44]	0.04	0.83
Infectious disease	1.13 [0.89–1.43]	0.98	0.32
Cognitive impairment	2.31 [1.74–3.06]	33.44	<0.001
Metabolic disorders	0.95 [0.73–1.25]	0.12	0.73
Total ED time (hours)	1.01 [0.99–1.01]	1.51	0.22

All-cause in-hospital death.

**Table 4:** Statistical comparison of surviving vs. deceased patients during hospitalization (all-cause death).

Variable	Alive n = 2226	Deceased n = 544	*p* Value
Age	84 [77–89]	86 [79–91]	<0.001
Sex (male)	925 (41.6)	263 (48.3)	0.002
Living Arrangements (% in rows)			
Living alone	485 (87.7)	68 (12.3)	
Living with partner	587 (84.8)	105 (15.2)	<0.001
Living with Relatives	790 (77.9)	224 (22.1)	
Nursing home resident	364 (71.2)	147 (28.8)	
Clinical scales			
Clinical Frailty Scale (CFS)	6 [5–7]	7 [7–8]	<0.001
Fit (CFS 1–3)	213 (9.6)	14 (2.6)	
Mild frailty (CFS 4–6)	981 (44.1)	122 (22.4)	<0.001
Frail (CFS 7–9)	1032 (46.4)	408 (75)	
Clinical Dementia Rating	1 [0–2]	2 [1–3]	<0.001
RASS value	0 [0–0]	−1 [−3–0]	<0.001
RASS-ABS *	0 [0–1]	1 [0–3]	<0.001
Charlson Comorbidity Index	5 [4–7]	6 [5–7]	<0.001
NEWS >5	303 (13.6)	134 (24.6)	
NEWS 3–5	825 (37.1)	216 (39.7)	<0.001
NEWS <3	1098 (49.3)	194 (35.7)	<0.001
ED Presentation symptoms			
Trauma	399 (17.9)	64 (11.8)	0.001
Dyspnea	496 (22.3)	135 (24.8)	0.2
Fever	673 (30.2)	185 (34)	0.09
Cough	86 (3.9)	17 (3.1)	0.25
Chest pain	83 (3.7)	9 (1.7)	0.02
Syncope	242 (10.9)	17 (3.1)	<0.001
Abdominal pain	228 (10.2)	40 (7.4)	0.04
Vomit	187 (8.4)	40 (7.4)	0.42
Pain (any)	118 (5.3)	36 (6.6)	0.23
Disorientation	309 (13.9)	60 (11)	0.08
Decreased consciousness	184 (8.3)	94 (17.3)	<0.001
Focal neurologic deficits	267 (12)	55 (10.1)	0.22
Malaise/fatigue	269 (12.1)	80 (14.7)	0.06
Symptom onset (>24 h)	991 (44.5)	251 (46.1)	0.26
Vitals			
Heart rate	85 [75–98]	86 [75–100]	0.049
Systolic blood pressure	130 [110–150]	120 [100–140]	<0.001
Diastolic blood pressure	75 [65–86]	70 [60–81]	<0.001
SpO2	96 [94–98]	95 [92–97]	<0.001
Body temperature	36.2 [36–36.8]	36 [36–36.9]	0.19
Laboratory values			
Serum creatinine	0.93 [0.73–1.41]	1.11 [0.74–1.96]	<0.001
Glucose	119 [101–153]	124 [103–148]	0.44
NTproBNP	2471 [981–6770]	4706 [1235–12,478]	0.001
Hb	12 [10.5–13.4]	12 [10.3–13.6]	0.68
WBC	9.65 [7.26–12.9]	11 [8–16.2]	<0.001
PLT	242 [180–319]	210 [151–308]	<0.001
C-reactive protein	28 [7–102]	68 [29–151]	
Procalcitonin	0.12 [0.05–0.51]	0.22 [0.08–0.76]	<0.001
Clinical history			
Ischemic heart disease	198 (8.9)	49 (9)	0.93
Congestive heart failure	615 (27.6)	186 (34.2)	0.002
Atrial fibrillation	429 (19.3)	90 (16.5)	0.14
Peripheral vascular disease	79 (3.5)	16 (2.9)	0.48
Previous stroke	224 (10.1)	84 (15.4)	<0.001
Dementia	754 (33.9)	227 (41.7)	0.001
COPD ^#^	279 (12.5)	69 (12.7)	0.92
Diabetes	308 (13.8)	72 (13.2)	0.71
Liver chronic disease	110 (4.9)	33 (6.1)	0.28
Chronic kidney disease	315 (14.2)	136 (25)	<0.001
Malignancy	323 (14.5)	96 (17.6)	0.07
Palliative care	28 (1.3)	12 (2.2)	0.1
Discharge diagnosis group			
Cardiovascular disease	532 (23.9)	111 (20.4)	
Fall/trauma	148 (6.6)	18 (3.3)	
Infectious disease	667 (30)	190 (34.9)	0.01
Cognitive impairment	285 (12.8)	75 (13.8)	
Malignancy	241 (10.8)	64 (11.8)	
Metabolic disorders	353 (15.9)	86 (15.8)	
Outcomes			
Delirium	675 (30.3)	308 (56.6)	<0.001
Total ED time (hours)	49.3 [28.2–75.7]	45.5 [26.7–70.2]	0.001
LOS ^£^ (days)	10 [7–16]	9 [4–18]	<0.001

* Richmond Agitation-Sedation Scale (RASS) absolute value; ^#^ chronic obstructive pulmonary disease; ^£^ length of hospital stay.

**Table 5:** Multivariate Cox regression analysis model for all causes of in-hospital death.

Variable	Hazard Ratio	Wald	*p*-Value
Age	1.03 [1.02–1.04]	39.871	<0.001
Sex (male)	1.51 [1.27–1.81]	20.778	<0.001
Clinical scales			
CFS	1.44 [1.34–1.55]	98.144	<0.001
CCI	1.02 [0.97–1.06]	0.857	0.355
NEWS > 5	Reference category	18.671	
NEWS 3–5	0.76 [0.60–0.94]	5.885	0.015
NEWS < 3	0.59 [0.47–0.76]	18.575	<0.001
Delirium onset in the ED	1.57 [1.30–1.88]	22.904	<0.001
Living Arrangements			
Living alone	Reference Category	11.333	
Living with partner	1.06 [0.77–1.45]	0.116	0.734
Living with relatives	1.41 [1.07–1.86]	5.809	0.016
Nursing home resident	1.03 [0.76–1.40]	0.004	0.825
Discharge diagnosis			
Cardiovascular disease	Reference Category	4.741	
Fall/trauma	0.78 [0.58–1.07]	2.232	0.127
Infectious disease	0.82 [0.49–1.35]	0.604	0.437
Cognitive impairment	1.01 [0.78–1.29]	0.004	0.952
Malignancy	0.99 [0.74–1.32]	0.003	0.958
Metabolic disorders	1.11 [0.78–1.56]	0.330	0.566
Total ED time (hours)	1.01 [0.99–1.01]	19.100	<0.001

## Data Availability

The data presented in this study are available upon reasonable requests made relative to the corresponding author.

## References

[1] Pallin D.J., Allen M.B., Espinola J.A., Camargo C.A., Bohan J.S. (2013). Population aging and emergency departments: Visits will not increase, lengths-of-stay and hospitalizations will. Health Aff..

[2] Preston L., van Oppen J.D., Conroy S.P., Ablard S., Buckley Woods H., Mason S.M. (2021). Improving outcomes for older people in the emergency department: A review of reviews. Emerg. Med. J..

[3] Šteinmiller J., Routasalo P., Suominen T. (2015). Older people in the emergency department: A literature review. Int. J. Older People Nurs..

[4] Dent E., Hoogendijk E.O., Cardona-Morrell M., Hillman K. (2016). Frailty in emergency departments. Lancet.

[5] Barrenetxea J., Tan K.B., Tong R., Chua K., Feng Q., Koh W.-P., Chen C. (2021). Emergency hospital admissions among older adults living alone in the community. BMC Health Serv. Res..

[6] Lee H., Lee E., Jang I.Y. (2020). Frailty and Comprehensive Geriatric Assessment. J. Korean Med. Sci..

[7] Vermeiren S., Vella-Azzopardi R., Beckwée D., Habbig A.-K., Scafoglieri A., Jansen B., Bautmans I., Gerontopole Brussels Study Group (2016). Frailty and the Prediction of Negative Health Outcomes: A Meta-Analysis. J. Am. Med. Dir. Assoc..

[8] Oliveira JESilva L., Stanich J.A., Jeffery M.M., Lindroth H.L., Miller D.M., Campbell R.L., Rabinstein A.A., Pignolo R.J., Bellolio F. (2022). Association between emergency department modifiable risk factors and subsequent delirium among hospitalized older adults. Am. J. Emerg. Med..

[9] Bellelli G., Triolo F., Ferrara M.C., Deiner S.G., Morandi A., Cesari M., Davis D., Marengoni A., Inzitari M., Watne L.O. (2024). Delirium and frailty in older adults: Clinical overlap and biological underpinnings. J. Intern. Med..

[10] Neufeld K.J., Thomas C. (2013). Delirium: Definition, epidemiology, and diagnosis. J. Clin. Neurophysiol..

[11] Zhang Z., Pan L., Ni H. (2013). Impact of delirium on clinical outcome in critically ill patients: A meta-analysis. Gen. Hosp. Psychiatry.

[12] Sachdev P.S., Blacker D., Blazer D.G., Ganguli M., Jeste D.V., Paulsen J.S., Petersen R.C. (2014). Classifying neurocognitive disorders: The DSM-5 approach. Nat. Rev. Neurol..

[13] Oliveira JESilva L., Berning M.J., Stanich J.A., Gerberi D.J., Murad M.H., Han J.H., Bellolio F. (2021). Risk Factors for Delirium in Older Adults in the Emergency Department: A Systematic Review and Meta-Analysis. Ann. Emerg. Med..

[14] Kennedy M., Enander R.A., Tadiri S.P., Wolfe R.E., Shapiro N.I., Marcantonio E.R. (2014). Delirium risk prediction, healthcare use and mortality of elderly adults in the emergency department. J. Am. Geriatr. Soc..

[15] Faheem W., Nandra T., Richardson S., Saliu D., Jackson T.A., Magill L., McCluskey L., Perry R., Welch C., Geriatric Medicine Research Collaborative (2023). Increasing frailty is associated with higher prevalence and reduced recognition of delirium in older hospitalised inpatients: Results of a multi-centre study. Eur. Geriatr. Med..

[16] Rockwood K., Song X., MacKnight C., Bergman H., Hogan D.B., McDowell I., Mitnitski A. (2005). A global clinical measure of fitness and frailty in elderly people. CMAJ.

[17] Lin L., Peng Y., Zhang H., Huang X., Chen L., Lin Y. (2022). Family-centred care interventions to reduce the delirium prevalence in critically ill patients: A systematic review and meta-analysis. Nurs. Open.

[18] Mohsen S., Moss S.J., Lucini F., Krewulak K.D., Stelfox H.T., Niven D.J., Sauro K.M., Fiest K.M. (2022). Impact of Family Presence on Delirium in Critically Ill Patients: A Retrospective Cohort Study. Crit. Care Med..

[19] Carbone M.K., Gugliucci M.R. (2015). Delirium and the Family Caregiver: The Need for Evidence-based Education Interventions. Gerontologist.

[20] Rieck K.M., Pagali S., Miller D.M. (2020). Delirium in hospitalized older adults. Hosp. Pract..

[21] Hofman M.R., van den Hanenberg F., Sierevelt I.N., Tulner C.R. (2017). Elderly patients with an atypical presentation of illness in the emergency department. Neth. J. Med..

[22] Jarrett P.G., Rockwood K., Carver D., Stolee P., Cosway S. (1995). Illness presentation in elderly patients. Arch. Intern. Med..

[23] Berman P., Hogan D.B., Fox R.A. (1987). The atypical presentation of infection in old age. Age Ageing.

[24] Limpawattana P., Phungoen P., Mitsungnern T., Laosuangkoon W., Tansangworn N. (2016). Atypical presentations of older adults at the emergency department and associated factors. Arch. Gerontol. Geriatr..

[25] O’Shaughnessy Í., Romero-Ortuno R., Edge L., Dillon A., Flynn S., Briggs R., Shields D., McMahon G., Hennessy A., Kennedy U. (2021). Home FIRsT: Interdisciplinary geriatric assessment and disposition outcomes in the Emergency Department. Eur. J. Intern. Med..

[26] Southerland L.T., Hunold K.M., Van Fossen J., Caterino J.M., Gulker P., Stephens J.A., Bischof J.J., Farrell E., Carpenter C.R., Mion L.C. (2022). An implementation science approach to geriatric screening in an emergency department. J. Am. Geriatr. Soc..

[27] American College of Emergency Physicians (2013). Geriatric Emergency Department Guidelines.

[28] Salini S., Giovannini S., Covino M., Barillaro C., Acampora N., Gravina E.M., Loreti C., Damiano F.P., Franceschi F., Russo A. (2022). Frailty Network in an Acute Care Setting: The New Perspective for Frail Older People. Diagnostics.

[29] Seidenfeld J., Lee S., Ragsdale L., Nickel C.H., Liu S.W., Kennedy M. (2024). Risk factors and risk stratification approaches for delirium screening: A Geriatric Emergency Department Guidelines 2.0 systematic review. Acad. Emerg. Med..

[30] Smith G.B., Prytherch D.R., Meredith P., Schmidt P.E., Featherstone P.I. (2013). The ability of the National Early Warning Score (NEWS) to discriminate patients at risk of early cardiac arrest, unanticipated intensive care unit admission, and death. Resuscitation.

[31] Kaeppeli T., Rueegg M., Dreher-Hummel T., Brabrand M., Kabell-Nissen S., Carpenter C.R., Bingisser R., Nickel C.H. (2020). Validation of the Clinical Frailty Scale for Prediction of Thirty-Day Mortality in the Emergency Department. Ann. Emerg. Med..

[32] Baten V., Busch H.-J., Busche C., Schmid B., Heupel-Reuter M., Perlov E., Brich J., Klöppel S. (2018). Validation of the Brief Confusion Assessment Method for Screening Delirium in Elderly Medical Patients in a German Emergency Department. Acad. Emerg. Med..

[33] Sessler C.N., Gosnell M.S., Grap M.J., Brophy G.M., O’Neal P.V., Keane K.A., Tesoro E.P., Elswick R.K. (2002). The Richmond Agitation-Sedation Scale: Validity and reliability in adult intensive care unit patients. Am. J. Respir. Crit. Care Med..

[34] Morris J.C. (1997). Clinical dementia rating: A reliable and valid diagnostic and staging measure for dementia of the Alzheimer type. Int. Psychogeriatr..

[35] Charlson M., Szatrowski T.P., Peterson J., Gold J. (1994). Validation of a combined comorbidity index. J. Clin. Epidemiol..

[36] Lewis L.M., Miller D.K., Morley J.E., Nork M.J., Lasater L.C. (1995). Unrecognized delirium in ED geriatric patients. Am. J. Emerg. Med..

[37] Giroux M., Sirois M.-J., Boucher V., Daoust R., Gouin É., Pelletier M., Berthelot S., Voyer P., Émond M. (2018). Frailty Assessment to Help Predict Patients at Risk of Delirium When Consulting the Emergency Department. J. Emerg. Med..

[38] Bellelli P.G., Biotto M., Morandi A., Meagher D., Cesari M., Mazzola P., Annoni G., Zambon A. (2019). The relationship among frailty, delirium and attentional tests to detect delirium: A cohort study. Eur. J. Intern. Med..

[39] Choutko-Joaquim S., Tacchini-Jacquier N., Pralong D’Alessio G., Verloo H. (2019). Associations between Frailty and Delirium among Older Patients Admitted to an Emergency Department. Dement. Geriatr. Cogn. Disord. Extra.

[40] O’Regan N.A., Ryan D.J., Boland E., Connolly W., McGlade C., Leonard M., Clare J., Eustace J.A., Meagher D., Timmons S. (2014). Attention! A good bedside test for delirium?. J. Neurol. Neurosurg. Psychiatry.

[41] Carpenter C.R., Shelton E., Fowler S., Suffoletto B., Platts-Mills T.F., Rothman R.E., Hogan T.M. (2015). Risk factors and screening instruments to predict adverse outcomes for undifferentiated older emergency department patients: A systematic review and meta-analysis. Acad. Emerg. Med..

[42] Mohammed M.A., Faisal M., Richardson D., Scally A., Howes R., Beatson K., Irwin S., Speed K. (2019). The inclusion of delirium in version 2 of the National Early Warning Score will substantially increase the alerts for escalating levels of care: Findings from a retrospective database study of emergency medical admissions in two hospitals. Clin. Med..

[43] Covino M., Sandroni C., Della Polla D., De Matteis G., Piccioni A., De Vita A., Russo A., Salini S., Carbone L., Petrucci M. (2023). Predicting ICU admission and death in the Emergency Department: A comparison of six early warning scores. Resuscitation.

[44] Joseph J.W., Elhadad N., Mattison M.L.P., Nentwich L.M., Levine S.A., Marcantonio E.R., Kennedy M. (2024). Boarding Duration in the Emergency Department and Inpatient Delirium and Severe Agitation. JAMA Netw. Open.

[45] Han J.H., Morandi A., Ely E.W., Callison C., Zhou C., Storrow A.B., Dittus R.S., Habermann R., Schnelle J. (2009). Delirium in the nursing home patients seen in the emergency department. J. Am. Geriatr. Soc..

[46] Khan I., Khan M.A. (2024). Sensory and Perceptual Alterations.

[47] Stollings J.L., Kotfis K., Chanques G., Pun B.T., Pandharipande P.P., Ely E.W. (2021). Delirium in critical illness: Clinical manifestations, outcomes, and management. Intensiv. Care Med..

[48] Khalsa S.S., Adolphs R., Cameron O.G., Critchley H.D., Davenport P.W., Feinstein J.S., Feusner J.D., Garfinkel S.N., Lane R.D., Mehling W.E. (2018). Interoception and Mental Health: A Roadmap. Biol. Psychiatry Cogn. Neurosci. Neuroimaging.

[49] Brewer R., Murphy J., Bird G. (2021). Atypical interoception as a common risk factor for psychopathology: A review. Neurosci. Biobehav. Rev..

[50] Mahler K.J. (2016). The Comprehensive Assessment for Interoceptive Awareness.

[51] Murphy J., Brewer R., Catmur C., Bird G. (2017). Interoception and psychopathology: A developmental neuroscience perspective. Dev. Cogn. Neurosci..

[52] Vrettos I., Voukelatou P., Panayiotou S., Kyvetos A., Tsigkri A., Makrilakis K., Sfikakis P.P., Niakas D. (2022). Factors Associated With Mortality in Elderly Hospitalized Patients at Admission. Cureus.

[53] Boonmee P., Ruangsomboon O., Limsuwat C., Chakorn T. (2020). Predictors of Mortality in Elderly and Very Elderly Emergency Patients with Sepsis: A Retrospective Study. West. J. Emerg. Med..

[54] Elliott A., Taub N., Banerjee J., Aijaz F., Jones W., Teece L., van Oppen J., Conroy S. (2021). Does the Clinical Frailty Scale at Triage Predict Outcomes From Emergency Care for Older People?. Ann. Emerg. Med..

[55] Ellis G., Marshall T., Ritchie C. (2014). Comprehensive geriatric assessment in the emergency department. Clin. Interv. Aging.

[56] Covino M., Salini S., Russo A., De Matteis G., Simeoni B., Maccauro G., Sganga G., Landi F., Gasbarrini A., Franceschi F. (2022). Frailty Assessment in the Emergency Department for Patients ≥ 80 Years Undergoing Urgent Major Surgical Procedures. J. Am. Med. Dir. Assoc..

[57] Rosa F., Covino M., Russo A., Salini S., Forino R., Della Polla D., Fransvea P., Quero G., Fiorillo C., La Greca A. (2022). Frailty assessment as independent prognostic factor for patients ≥ 65 years undergoing urgent cholecystectomy for acute cholecystitis. Dig. Liver Dis..

[58] Inouye S.K., Bogardus S.T., Charpentier P.A., Leo-Summers L., Acampora D., Holford T.R., Cooney L.M.J. (1999). A multicomponent intervention to prevent delirium in hospitalized older patients. N. Engl. J. Med..

[59] Ehrlich A., Oh E.S., Ahmed S. (2024). Managing Delirium in the Emergency Department: An Updated Narrative Review. Curr. Geriatr. Rep..

[60] Martínez F., Donoso A.M., Marquez C., Labarca E. (2017). Implementing a Multicomponent Intervention to Prevent Delirium Among Critically Ill Patients. Crit. Care Nurse.

[61] Qin M., Gao Y., Guo S., Lu X., Zhu H., Li Y. (2022). Family intervention for delirium for patients in the intensive care unit: A systematic meta-analysis. J. Clin. Neurosci..

[62] Chen T.-J., Traynor V., Wang A.-Y., Shih C.-Y., Tu M.-C., Chuang C.-H., Chiu H.-Y., Chang H.-C.R. (2022). Comparative effectiveness of non-pharmacological interventions for preventing delirium in critically ill adults: A systematic review and network meta-analysis. Int. J. Nurs. Stud..

